# The Elevated-Temperature Nano-Mechanical Properties of a PDMS–Silica-Based Superhydrophobic Nanocomposite Coating

**DOI:** 10.3390/nano15120898

**Published:** 2025-06-10

**Authors:** Chun-Wei Yao, Ian Lian, Jiang Zhou, Paul Bernazzani, Mien Jao

**Affiliations:** 1 Department of Mechanical Engineering, Lamar University, Beaumont, TX 77710, USA; zhoujx@lamar.edu; 2 Department of Biology, Lamar University, Beaumont, TX 77710, USA; ilian@lamar.edu; 3 Department of Chemistry and Biochemistry, Lamar University, Beaumont, TX 77710, USA; pbernazzan@lamar.edu; 4 Department of Civil and Environmental Engineering, Lamar University, Beaumont, TX 77710, USA; jaomu@lamar.edu

**Keywords:** nanocomposite, nanocreep, nanoscale dynamic mechanical analysis

## Abstract

This study investigates the elevated-temperature mechanical and viscoelastic properties of a PDMS–silica-based superhydrophobic nanocomposite coating using nanoindentation and a nano-dynamic mechanical analysis over a temperature range of 24 °C to 160 °C. The nanoindentation load–displacement curves exhibited consistent hysteresis, indicating a stable energy dissipation across the temperature range. Creep tests revealed an increased displacement and accelerated deformation at elevated temperatures, displaying a two-stage creep profile characterized by rapid primary and steady-state secondary creep. The hardness decreased with the creep time, while the strain rate sensitivity remained relatively stable, suggesting consistent deformation mechanisms. A time-dependent creep model incorporating linear and logarithmic terms accurately captured the experimental data. The nano-dynamic mechanical analysis results showed a decrease in the storage modulus with depth, while the loss modulus and tan δ peaked at shallow depths. These findings are crucial for the evaluation and design of superhydrophobic nanocomposite coatings.

## 1. Introduction

Engineered superhydrophobic surfaces, those with water contact angles greater than 150°, are specifically designed to reduce liquid adhesion. This allows water droplets to roll off easily, giving these surfaces self-cleaning properties [1,2]. This impressive behavior results from a combination of the surface’s natural hydrophobicity and its micro- or nanoscale texture, both of which are essential for an optimal function and durability [3]. Superhydrophobic nanocomposites are especially valuable due to their broad range of applications, including biomedical devices, environmental sensors, and protective coatings for infrastructure. These uses benefit from the materials’ enhanced resistance to contamination and corrosion [4,5]. There are several ways to create superhydrophobic coatings, such as using nanocomposite materials [6,7,8], chemical vapor deposition [9], self-assembled monolayers [10], and template-based methods [11].

Nanocomposites, which consist of nanoparticles dispersed within a polymer matrix, offer improved mechanical and chemical properties [12]. These coatings are particularly attractive because their characteristics can be easily tuned by adjusting the composition of the coating solution [13]. Importantly, nanocomposite coatings can achieve both key requirements for superhydrophobicity, surface roughness and a low surface energy, in a single fabrication step. Superhydrophobic coatings have attracted growing interest in the aerospace and marine industries due to their ability to reduce drag and resist icing. In these demanding environments, coatings must retain their functional properties under fluctuating thermal and mechanical loads, making thermal stability a key performance criterion. Incorporating silica nanoparticles into a PDMS not only improves the mechanical strength but also contributes to the hierarchical surface morphology required for superhydrophobicity.

Studying the nano-mechanical properties of these surfaces is crucial for developing advanced materials with better performances across a wide range of applications [14,15]. These characterization techniques help us better understand the micromechanics and strengthening mechanisms that affect nanocomposites. This knowledge supports the design of materials for use in everything from biomedical tools to structural components and everyday products [16,17]. One important aspect is nanocreep, which refers to the slow, time-dependent deformation of materials under a constant load at the nanoscale. Gaining insight into nanocreep is essential for improving the mechanical performance of nanocomposites, which often outperform traditional composites in terms of strength or thermal stability [12]. Temperature also plays a significant role, as it can greatly influence nanocreep behavior and reveal the complex interactions within these materials [18,19].

Recent research has used nanoindentation to study the mechanical and creep behavior of various materials. These studies have measured key mechanical properties and examined how the load and strain rate affect creep behavior [20,21,22,23,24]. Nanoindentation also provides precise measurements of the hardness and elastic modulus, which are essential for understanding creep. Meanwhile, a nano-dynamic mechanical analysis (nano-DMA) offers insights into how materials respond to dynamic loading conditions [14,25]. Both nanoindentation and nano-DMA are advanced techniques for evaluating the mechanical behavior of nanocomposites at very small scales, allowing researchers to explore the viscoelastic properties of nanocomposites [16,26].

In this study, the nanoscale mechanical response of a superhydrophobic nanocomposite coating subjected to elevated temperatures was investigated. Using nanoindentation techniques, parameters such as the nanohardness, strain rate sensitivity, and time-dependent creep behavior were quantified. Notably, the authors believe this is the first analysis of the coating’s viscoelastic properties, specifically the hardness, storage modulus, loss modulus, and tan δ at various temperatures. The results not only shed light on the material’s performance in elevated-temperature environments but also highlight its potential suitability for demanding industrial and engineering applications where both mechanical properties and thermal stability are critical.

## 2. Materials and Methods

Steel substrates were used as the base material. The chemical reagents included acetone, anhydrous ethanol, and isopropyl alcohol (Sigma Aldrich, St. Louis, MO, USA). A silane-modified hydrophobic SiO_2_ nanoparticle powder, RX-50 (Evonik, Piscataway, NJ, USA), with an average diameter of 55 ± 15 nm along with a polydimethylsiloxane (PDMS) elastomer kit (Sylgard 184, Dow Corning, Midland, MI, USA) were employed for coating preparation.

Before coating deposition, the substrates underwent a cleaning protocol involving degreasing followed by ultrasonic cleaning for 20 min at room temperature. The substrates were then sequentially rinsed with isopropyl alcohol, ethanol, and deionized (DI) water to remove surface contaminants.

The superhydrophobic nanocomposite coating solution was prepared through a multi-step process. First, 1.7 g of PDMS was dissolved in 11 g of toluene using an ultrasonic mixer for 1 min to achieve uniformity. Separately, 2 g of SiO_2_ nanoparticles were dispersed in 10 g of toluene using a planetary centrifugal mixer for 30 s to ensure a homogeneous suspension. These two mixtures were then combined and further homogenized using the same mixer for an additional 30 s. Next, 0.17 g of curing agent was added to the combined solution, followed by a final 30 s of mixing and 30 s of defoaming to eliminate entrapped air. The prepared nanocomposite coating was applied to the substrates using a spray-coating technique. Finally, the coated substrates were cured at ambient conditions (25 °C) inside a fume hood for 72 h to allow complete PDMS cross-linking. Toluene was selected as the solvent due to its compatibility with both PDMS and silica nanoparticles, facilitating uniform dispersion and minimizing premature agglomeration. The planetary centrifugal mixer was employed for its ability to generate high shear forces, effectively breaking up nanoparticle clusters and ensuring a homogeneous suspension.

The static contact angle was measured using a drop shape analyzer (DSA25E, Krüss, Matthews, NC, USA) by placing 10 µL deionized water droplets on the surface. Surface morphology was characterized using scanning electron microscopy (SEM, JSM-7500F, JEOL, Peabody, MA, USA), while surface topography was further examined with atomic force microscopy (AFM, Park NX10, Park Systems Co., Ltd., Santa Clara, CA, USA). Mechanical properties were evaluated through nanoindentation and nano-dynamic mechanical analysis (nano-DMA) using a TI 980 TriboIndenter (Bruker, Eden Prairie, MN, USA) equipped with a three-sided Berkovich diamond tip. All creep and nano-DMA tests were conducted under a maximum indentation load of 1500 μN. The coating thickness was measured using a digital coating thickness gauge (Elcometer, Manchester, UK).

## 3. Results

**Figure 1:**
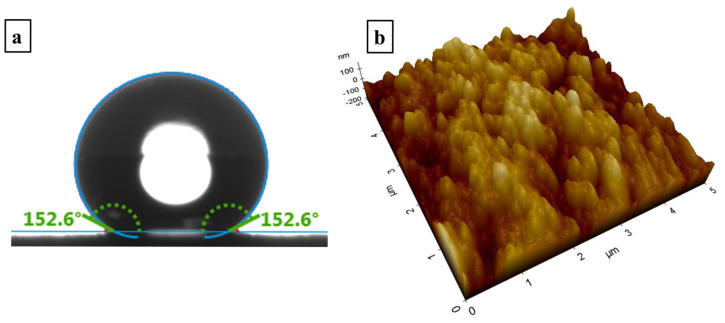
(**a**) A static contact angle image for a water droplet of 10 µL on the superhydrophobic nanocomposite coating; (**b**) an AFM image of the superhydrophobic nanocomposite coating.

Figure 1a shows the static contact angle measured on the superhydrophobic nanocomposite coating. The incorporation of silica nanoparticles within the PDMS matrix yielded a high static contact angle of 152.6°, demonstrating exceptional hydrophobicity.

**Figure 2:**
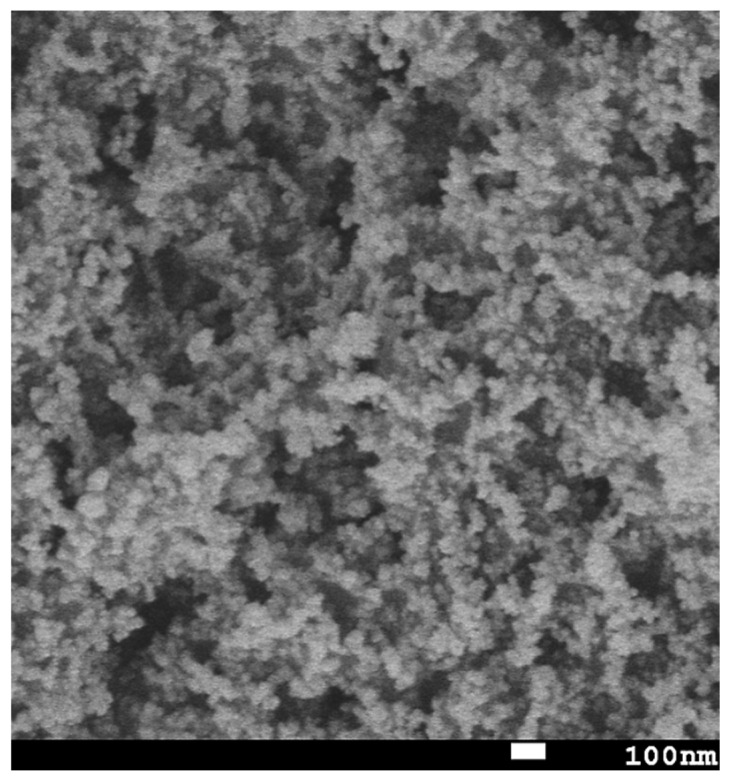
An SEM image of the superhydrophobic nanocomposite coating.

Figure 1b presents a three-dimensional atomic force microscopy (AFM) micrograph of the superhydrophobic nanocomposite coating. The observed hierarchical topography, characterized by the microscale roughness arising from the nanoparticle agglomeration and nanoscale features attributed to individual particles, significantly contributes to the enhanced hydrophobic properties. The scanning electron microscopy (SEM) image, depicted in Figure 2, reveals a distributed network of nanoparticles, further emphasizing the textured morphology of the resulting coating.

**Figure 3:**
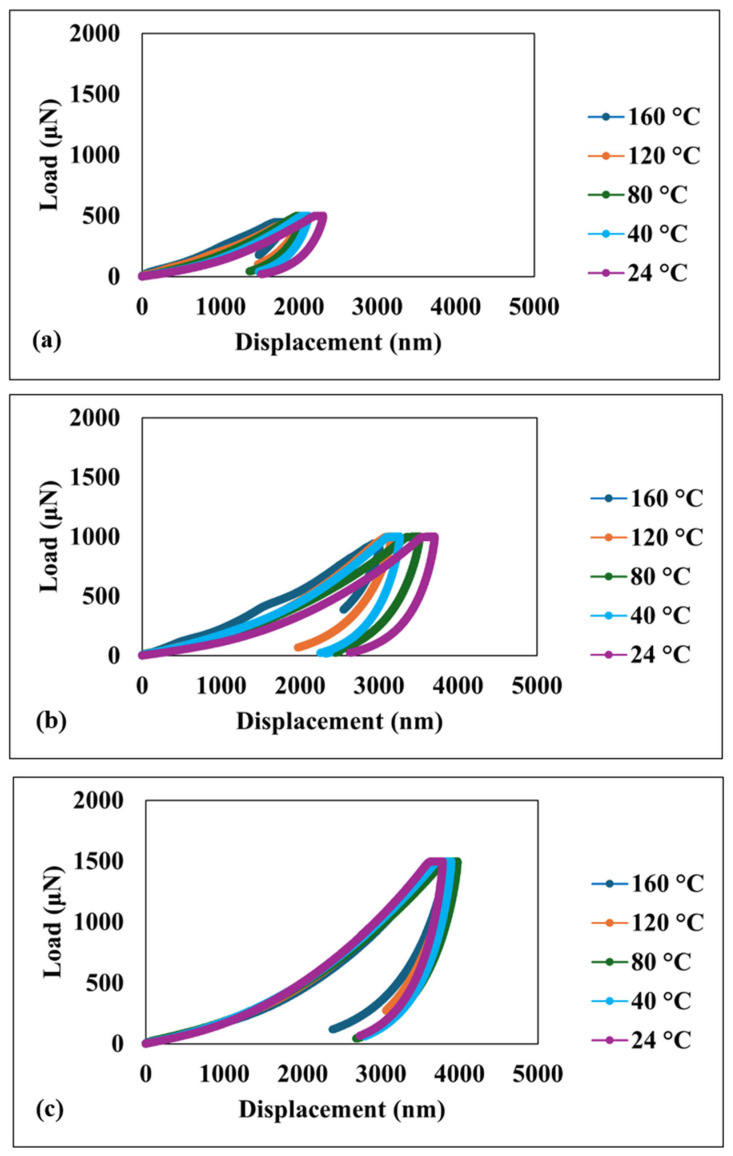
Load–displacement curves under different loads at various temperatures (**a**) 500 µN load; (**b**) 1000 µN load; (**c**) 1500 µN load.

Figure 3 shows the load–displacement curves obtained for the superhydrophobic nanocomposite coating at various temperatures. The depth of penetration is observed to increase with the applied load in all cases. Significantly, the hysteresis behavior exhibits consistency across the entire temperature range investigated, including the highest temperature of 160 °C. This consistency strongly suggests that the coating maintains its energy dissipation characteristics despite significant temperature variations.

**Figure 4:**
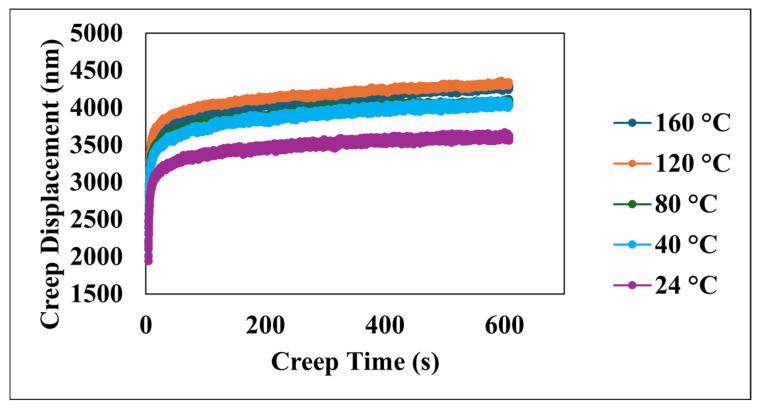
Creep displacement versus creep time at various temperatures.

Figure 4 illustrates the creep displacement of the material as a function of time, subjected to the constant applied load across a temperature range of 24 °C to 160 °C. The data clearly demonstrate a positive correlation between the increasing temperature and the greater creep displacement, with the maximum total creep displacement reaching approximately 4300 nm around 600 s, observed at both the highest test temperature of 160 °C and the 120 °C test, while the minimum creep displacement of approximately 3600 nm at the same time point corresponds to the lowest temperature of 24 °C. Furthermore, the rate of creep, as indicated by the slope of the displacement–time curves, exhibits a strong temperature dependency, where higher temperatures lead to a more pronounced initial increase in displacement, which is particularly evident within the initial 100 s; this observation underscores the significant influence of temperature in accelerating the underlying creep deformation mechanisms within the material, as all creep−time curves show a two-stage upward trend: an initial rapid rise within the first ~100 s, which corresponds to the instable primary creep, followed by a slow linear increase, which corresponds to a more stable secondary creep.

**Figure 5:**
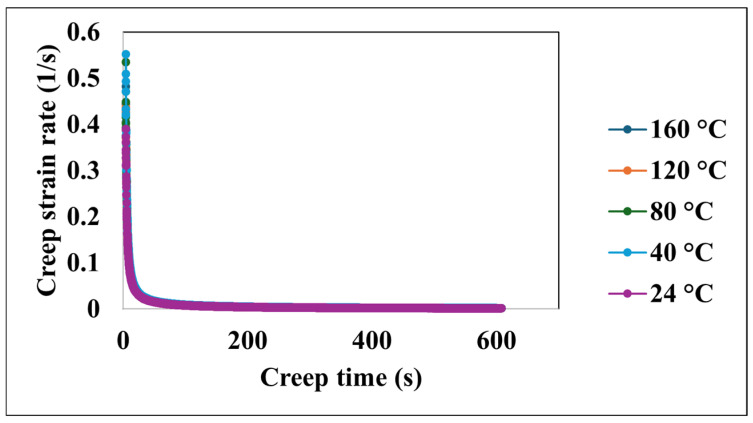
Creep strain rate versus creep time at various temperatures.

Figure 5 presents the evolution of the creep strain rate as a function of the creep time across the temperature range. An initial period of transient creep is evident, characterized by a rapid decrease in the creep strain rate at all investigated temperatures. Following this transient stage, the creep strain rate approaches a steady-state, nearly constant value. This behavior suggests significant initial deformation followed by a period of stable creep.

**Figure 6:**
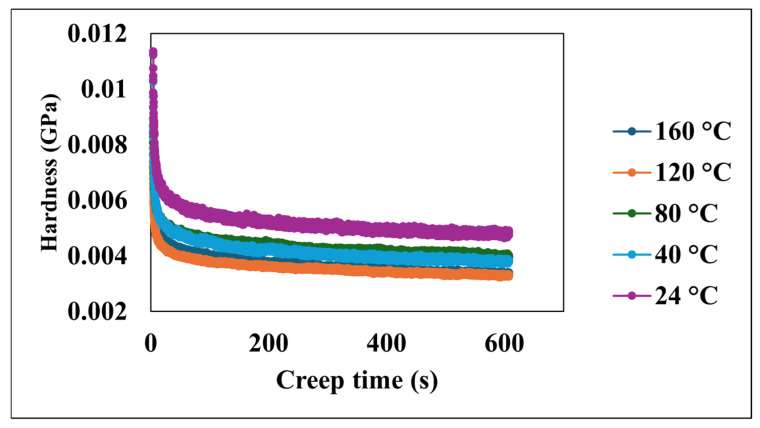
Hardness versus creep time at various temperatures.

Figure 6 presents the relationship between the creep time and hardness at various temperatures. Across all investigated temperatures, the hardness of the material consistently decreases as the creep time increases. Despite these variations in initial conditions, the universal trend of a decreasing hardness with time is evident for all temperatures. The observed decrease in hardness with the increasing creep time is consistent with the viscoelastic relaxation behavior characteristic of PDMS-based systems. This time-dependent softening in polymer nanocomposites under sustained loading is attributed to the gradual rearrangement of polymer chains and the redistribution of internal stresses.

The material’s sensitivity to changes in the strain rate during creep, quantified by the creep strain rate sensitivity (m), was analyzed using the logarithmic relationship between the stress and strain rate [27,28]:

log(stress) = (1/m) × log(strain rate) + a1(1)

where a1 is the fitting coefficient.

**Figure 7:**
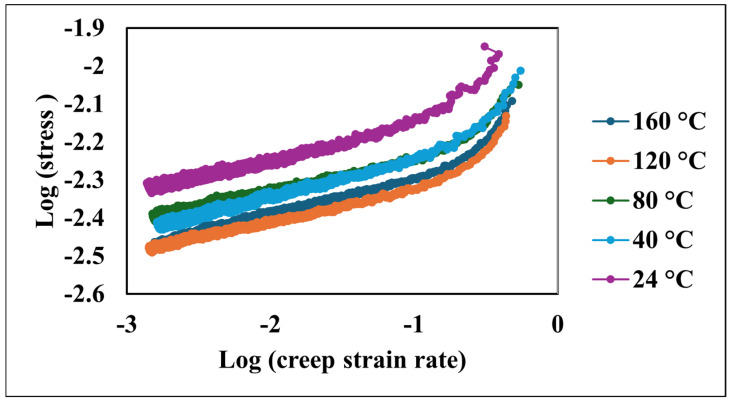
Logarithmic stress versus logarithmic creep strain rate at various temperatures.

Logarithmic plots of stress versus the creep strain rate demonstrated a robust linear correlation. As depicted in Figure 7, elevated temperatures corresponded to a shift in these curves towards higher stain rates at a given stress level. Notably, the slopes of these plots, which represent the strain rate sensitivity (m), exhibited minimal variation across the tested temperature spectrum, as shown in Table 1.

**Figure 8:**
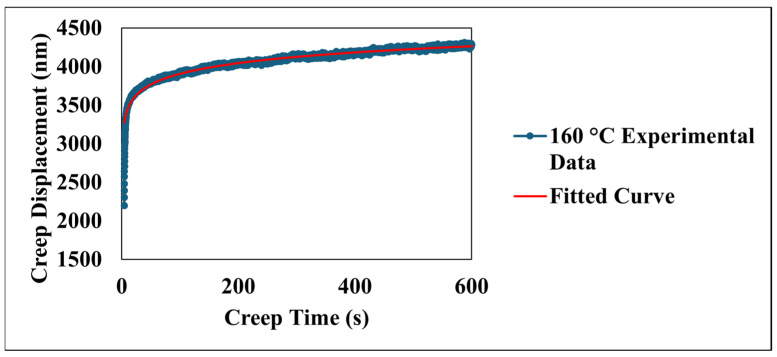
Experimental and fitted creep displacement behavior at selected temperature.

Figure 8 details the time-dependent creep displacement of the material under a sustained load at an elevated temperature of 160 °C. The experimental data, presented as a blue line, reveals a characteristic creep profile over the 600 s indentation period. Initially, a pronounced primary creep regime is evident within the first few hundred seconds, characterized by a rapid accumulation of creep displacement. This initial phase reflects the material’s immediate response to the applied stress, involving mechanisms such as elastic deformation. As time progresses, the rate of the creep displacement demonstrably diminishes, transitioning into a secondary, or steady-state, creep regime. This stage, occurring throughout the latter part of the 600 s test, exhibits a more gradual increase in displacement with time. As shown in Figure 8, the red line represents the fitted curve based on the model [29,30]:

creep displacement = a + bt + cln(dt + 1)(2)

where t is the creep time in seconds, and a, b, c, and d are the fitting coefficients shown in Table 2.

**Figure 9:**
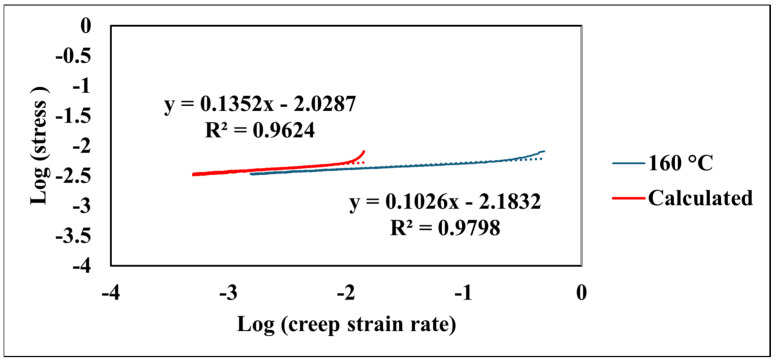
Comparison of experimental and calculated creep strain rate at selected temperature.

The model yielded a high correlation coefficient (R^2^) of 0.96, indicating a strong agreement between the model and the experimental data. The indentation test was concluded at 600 s, capturing the material’s behavior through the primary and well-established secondary creep stages, which are well-represented by the chosen model. The coefficients a, b, c, and d derived from this fitting procedure quantify different aspects of the creep behavior: coefficient a represents the initial displacement, coefficient b represents the linear contribution to creep over time (steady-state creep rate), coefficient c represents the magnitude of the logarithmic term capturing the primary creep behavior, and coefficient d represents the rate at which the logarithmic term influences the displacement. These parameters provide valuable insights into the material’s inherent resistance to creep and its sensitivity to the applied stress at 160 °C, contributing significantly to a comprehensive understanding of its elevated-temperature mechanical behavior. Furthermore, the efficacy of the model demonstrates its capability in capturing the creep behavior of the material across a range of temperatures from 24 °C to 160 °C. As presented in Table 2, the model consistently exhibits high correlation coefficients (R^2^) exceeding 0.94 for all tested temperatures. This strong agreement between the model’s results and the experimental data underscores the model’s ability to effectively describe the fundamental creep mechanisms of the coating. The variations observed in the coefficients a, b, c, and d with changing temperatures provide valuable insights into the temperature-dependent nature of these mechanisms. For instance, parameter a generally shows an increasing trend with temperature, suggesting a greater instantaneous deformation at elevated temperatures. Similarly, the evolution of parameter b, indicative of the steady-state creep rate, and the parameters c and d, associated with the primary creep regime, reflect the accelerated kinetics of deformation processes at higher temperatures. Figure 9 compares the experimentally observed relationship between the logarithm of the creep strain rate and the logarithm of stress with the values derived from the fitted model at an elevated temperature of 160 °C. The data derived from the model, depicted by the red line, is described by the equation log(creep strain rate) = 0.1352 × log(stress) − 2.0287 and also demonstrates a strong correlation coefficient (R^2^ = 0.96). The close values of the strain rate sensitivity obtained from the experimental data (m = 0.1026) and the fitted model (m = 0.1352) indicate that the model effectively captures the material’s sensitivity to changes in applied stress.

**Figure 10:**
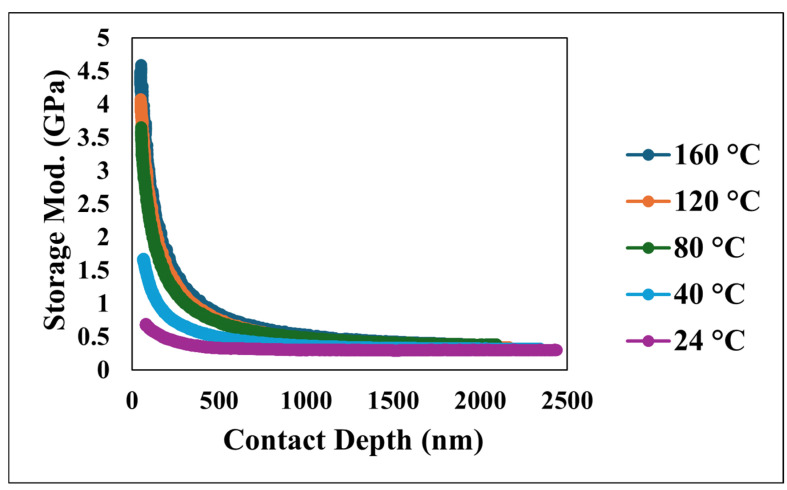
Storage modulus versus contact depth at various temperatures.

**Figure 11:**
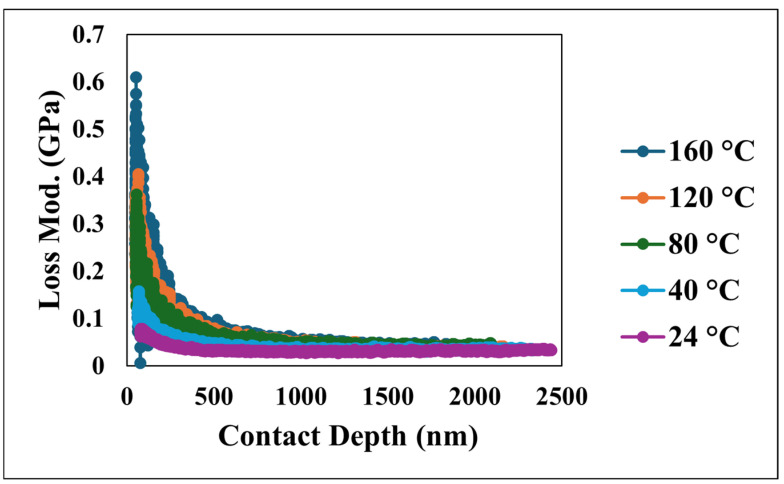
Loss modulus versus contact depth at various temperatures.

**Figure 12:**
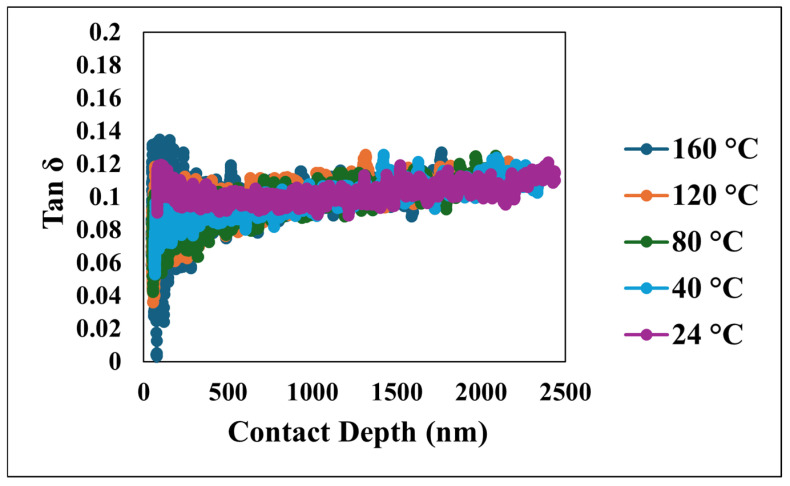
Tan δ versus contact depth at various temperatures.

The nano-dynamic mechanical analysis (nano-DMA) provided a detailed characterization of the viscoelastic behavior of the superhydrophobic nanocomposite coating, with Figure 10, Figure 11 and Figure 12 presenting an overview of the storage modulus, loss modulus, and tan δ as a function of both the penetration depth and temperature. Specifically, Figure 10 illustrates the relationship between the storage modulus, a fundamental indicator of the material’s capacity to store elastic energy, and the contact depth at various temperatures. The data demonstrates a consistent trend of a decreasing storage modulus with an increasing contact depth across all tested temperatures. At the highest tested temperature of 160 °C, the storage modulus exhibited a considerably steeper decline as the indenter penetrated deeper into the material compared to the more gradual decrease observed at lower temperatures such as 24 °C. This contrast underscores a significant sensitivity of the material’s storage modulus to temperature variations.

Furthermore, the viscoelastic energy dissipation characteristics of the coating were investigated through the loss modulus and tan δ, depicted in Figure 11 and Figure 12, respectively. The loss modulus, which quantifies the energy dissipated during the cyclic deformation, initially exhibited a peak which reflects the interactions occurring within the near-surface layers under dynamic loading. Following this initial value, the loss modulus consistently decreased and eventually stabilized at more constant values as the penetration depth increased, indicating a more homogeneous energy dissipation behavior. Similarly, tan δ, the ratio of the loss modulus to the storage modulus, which serves as a measure of the material’s damping capacity, mirrored the trend observed in the loss modulus. Tan δ initially showed an increase at shallow depths, which is indicative of significant damping near the surface, followed by a decrease and stabilization at greater depths. The initially elevated values of both the loss modulus and tan δ at shallow depths highlight the significant contribution of the unique nano/microstructural features of the superhydrophobic nanocomposite coating’s surface to its viscoelastic behavior, particularly in terms of energy dissipation and damping. However, as the indenter probes deeper, the material’s response becomes more uniform, reflecting the bulk viscoelastic properties.

## 4. Conclusions

In this study, the elevated-temperature mechanical and viscoelastic behavior of a superhydrophobic nanocomposite coating was analyzed using nanoindentation and a nano-DMA from 24 °C to 160 °C. The consistent hysteresis observed in the nanoindentation confirmed stable energy dissipation across this range. Temperature-dependent creep testing revealed an increased displacement and accelerated deformation at elevated temperatures, exhibiting a distinct two-stage profile. The strain rate sensitivity suggested consistent deformation mechanisms. A time-dependent creep model accurately predicted experimental data, with parameters reflecting enhanced creep kinetics at higher temperatures, which is crucial for predicting performance. The nano-DMA further exposed the coating’s viscoelastic response, showing an effective energy dissipation near the surface. Furthermore, the integration of the experimental nanoindentation data with a robust time-dependent creep model provides a powerful tool for predicting the mechanical behavior of superhydrophobic coatings in thermally demanding environments. The findings of this study highlight the potential of PDMS–silica nanocomposites for high-performance applications, where both thermal resilience and mechanical performance are critical. The validated creep model and the comprehensive viscoelastic characterization offer a solid foundation for future research and development in this field.

## Figures and Tables

**Table 1:** The coefficients for Equation (1).

Coefficient	1/m	a1	R^2^
24 °C case	0.09	−2.06	0.95
40 °C case	0.10	−2.14	0.97
80 °C case	0.08	−2.16	0.96
120 °C case	0.09	−2.24	0.97
160 °C case	0.10	−2.18	0.98

**Table 2:** The coefficients for the model.

Coefficient	a	b	c	d	R^2^
24 °C case	1044	−0.2016	187.1	2699	0.94
40 °C case	1078	−0.1581	215	2479	0.95
80 °C case	1257	−0.1641	201.1	3038	0.95
120 °C case	1618	−0.1051	190.6	3013	0.94
160 °C case	1359	−0.0008	199.6	3517	0.96

## Data Availability

The original contributions presented in this study are included in the article; further inquiries can be directed to the corresponding author.
